# Parameter selection for and implementation of a web-based decision-support tool to predict extubation outcome in premature infants

**DOI:** 10.1186/1472-6947-6-11

**Published:** 2006-03-01

**Authors:** Martina Mueller, Carol L Wagner, David J Annibale, Rebecca G Knapp, Thomas C Hulsey, Jonas S Almeida

**Affiliations:** 1 Department of Biostatistics, Bioinformatics & Epidemiology, Medical University of South Carolina, Charleston, SC, USA; 2 Department of Pediatrics, Medical University of South Carolina, Charleston, SC, USA; 3 Department of Biostatistics and Applied Mathematics, University of Texas MD Anderson Cancer Center, Houston TX, USA

## Abstract

**Background:**

Approximately 30% of intubated preterm infants with respiratory distress syndrome (RDS) will fail attempted extubation, requiring reintubation and mechanical ventilation. Although ventilator technology and monitoring of premature infants have improved over time, optimal extubation remains challenging. Furthermore, extubation decisions for premature infants require complex informational processing, techniques implicitly learned through clinical practice. Computer-aided decision-support tools would benefit inexperienced clinicians, especially during peak neonatal intensive care unit (NICU) census.

**Methods:**

A five-step procedure was developed to identify predictive variables. Clinical expert (CE) thought processes comprised one model. Variables from that model were used to develop two mathematical models for the decision-support tool: an artificial neural network (ANN) and a multivariate logistic regression model (MLR). The ranking of the variables in the three models was compared using the Wilcoxon Signed Rank Test. The best performing model was used in a web-based decision-support tool with a user interface implemented in Hypertext Markup Language (HTML) and the mathematical model employing the ANN.

**Results:**

CEs identified 51 potentially predictive variables for extubation decisions for an infant on mechanical ventilation. Comparisons of the three models showed a significant difference between the ANN and the CE (p = 0.0006). Of the original 51 potentially predictive variables, the 13 *most predictive *variables were used to develop an ANN as a web-based decision-tool. The ANN processes user-provided data and returns the prediction 0–1 score and a novelty index. The user then selects the most appropriate threshold for categorizing the prediction as a success or failure. Furthermore, the novelty index, indicating the similarity of the test case to the training case, allows the user to assess the confidence level of the prediction with regard to how much the new data differ from the data originally used for the development of the prediction tool.

**Conclusion:**

State-of-the-art, machine-learning methods can be employed for the development of sophisticated tools to aid clinicians' decisions. We identified numerous variables considered relevant for extubation decisions for mechanically ventilated premature infants with RDS. We then developed a web-based decision-support tool for clinicians which can be made widely available and potentially improve patient care world wide.

## Background

Approximately 470,000 infants (~12%) are born prematurely in the US each year. Virtually all infants born at ≤27 weeks gestation, ~80% of those delivered between 27 and 30 weeks' gestation, and approximately 30% of the infants born between 30 and 32 weeks' of gestation [1] will develop respiratory distress syndrome (RDS) requiring endotracheal intubation and intermittent positive pressure ventilation starting soon after birth. Nearly 30% of intubated preterm infants will fail attempted extubation, requiring reintubation and resumption of mechanical ventilation [2]. Over the past years, several attempts have been made to identify variables that are predictors for extubation outcome in premature infants with RDS. Although some studies [2-6] reported significant differences for certain variables between infant groups with either failed or successful extubations, to date no universally applicable model has been developed to predict extubation outcomes. Sample sizes used in these studies were usually small (range: 18–36). Some of the studies focused mainly on a small selected number of variables. Additionally, statistical analyses included t-tests, ANOVA, Mann-Whitney, and χ^2 ^tests, but no predictive models were developed. The problem of determining the optimal extubation time point for premature infants on artificial ventilation, however, is extremely complex. It is based not only on apparent relationships, e.g. blood gases and ventilator settings, but also on a large amount of information that is, in part, processed implicitly as the result of learning through clinical experience. Additionally, the decision whether or not to extubate depends on a large amount of information considered and weighed by the clinician.

Therefore, predicting the ideal time point for extubation in this population requires excellent diagnostic skills but remains a difficult task despite many technological advances over the past decade. Determining the optimal time point for extubation is crucial to minimize the infants' times on artificial ventilation, thus minimizing their risk of developing volutrauma (caused by large volumes in the lung) and barotrauma (caused by high pressures in the lung), retinopathy (caused by high arterial oxygen), infection and subsequent bronchopulmonary dysplasia (BPD) or chronic lung disease (CLD) [7]. The decision to extubate, however, is complicated by the smaller risk associated with too early extubation, having to reintubate, therefore subjecting the infants to subsequent increases of ventilatory support due to alveolar collapse or atelectasis that has occurred in the ensuing hours after extubation [8]. These risks could be reduced if an automated prediction system was available to assist the neonatal intensive care unit (NICU) staff with extubation decisions. Artificial neural networks (ANN) have been found to be effective in the prediction of extubation outcomes in adults [9,10]; however, only recently has the first use of an ANN been published for the prediction of extubation outcomes in premature infants by the authors [11].

In recent years, more medical tools have been developed and made available through the World Wide Web (Internet). Many of those tools were created for educational and research purposes, such as databases for evidence-based medicine, drug information databases, or learning tools, however, additional decision-support tools that aid clinicians in their decision-making are needed. Such tools would greatly benefit from similar widespread availability and maintenance-free, user-friendly interfaces.

Specifically, such decision-support tools would be particularly valuable during the care and treatment of premature infants in the NICU for the purpose of informing inexperienced clinicians about infants potentially ready for extubation during times when the NICU is overcrowded and extremely busy and these infants may only receive sporadic attention. Better infant intubation management would lower the infant's risk for re-intubation, prolonged ventilation, and associated risks, especially CLD, which in turn can reduce the number of days spent in the NICU for the infants, leading to substantial reductions in health care costs. Rogowski *et al*. [12] reported median treatment cost per infant in the NICU in 1994 of $85,959–$91,969 for infants with birth weights of 501–1,000 g and gestational age ≤34 weeks. In 1999, charges associated with specific perinatal diagnoses were reported to be the highest in health care; respiratory distress syndrome being the most expensive with a mean charge of $82,648 and a mean length of stay in the NICU of 27.8 days (Healthcare Utilization Project Nationwide Inpatient Sample, 1999, prepared by March of Dimes Perinatal Data Center, 2002). Similarly, health care savings have been demonstrated by improving extubation outcomes in mechanically ventilated adults by Ely *et al. *[13].

An artificial intelligence machine-learning approach for predicting extubation outcomes to support clinicians in their decision-making has been developed with data from 183 preterm newborns with RDS on mechanical ventilation [11]. Because clinicians using a decision-support tool would need to access the reliability of the individual predictions, measures of error such as receiver operating characteristic (ROC) curves and relevance such as a novelty index are provided. Consequently, the decision-support tool specifically can decrease the number of false-positive (i.e. infants who were extubated too early) and false-negative (infants who could have been extubated earlier) cases, potentially reducing the risks to intubated infants and the dollar costs accompanying higher acuity and extended lengths of hospitalization associated with these risks.

To capture all variables relevant to the decision-making, a paradigm was developed for environmental problems to delineate the use of uncertainties in risk assessment [14]. In these guidelines Haimes *et al. *suggested working closely with the decision-makers to convey important issues and further recommended the use of guiding questions as an important strategy.

In this report, we describe the modeling of the thought processes underlying infant extubation decisions and the multiple variable associated with these thought processes. Furthermore, we describe the development of a web-based tool to predict extubation outcome using the previously identified variables. This tool will provide clinicians with easy-to-use and widely accessible decision-support in the care of premature infants with RDS on mechanical ventilators.

## Methods

### Variable identification

Although risk assessment is mostly used for environmental problems, some of the guidelines proved useful in the identification of relevant variables for the prediction problem examined in this study [14]. Working closely with the decision-makers was translated into working closely with the clinicians to extract important information (i.e. which variables were used in their decision). In this work to identify potentially predictive parameters, the authors included 2 clinicians (CW, DA) for their expert opinion to establish a framework for the selection of the variables. Different experts, i.e. different neonatologists, may reach different conclusions, which may result from different levels of knowledge and expertise and different underlying assumptions. These different approaches are applied to patients with a variable amount of information, starting from background information about the infant, the pregnancy and delivery, and maternal health to the most current information about the status of the infant.

The development of guiding questions for this study as suggested by Haimes *et al. *[14] resulted in a 5-step procedure consisting of: 1) literature research and variable selection; 2) discussion resulting in discarding of variables; 3) panel discussion and adding of variables; 4) bedside visits and adding of variables; and 5) discussion about discarding of variables and inclusion of new variables.

#### 1) Literature research and variable selection

As a first step, a literature review (Medline) was carried out to identify variables that had been examined previously for their predictive capabilities. Six studies [2-5,15,16] reporting prediction capabilities for some variables were used to compile a list of potentially predictive parameters. A panel of experienced clinicians (CW, DA) then reviewed the list and discarded variables that were not available at the bedside at all times or that required additional examinations of the infant. This decision was made for two reasons. First, the study was based on retrospective chart review, and therefore, could only retrieve routine parameters of the infant's care in sufficiently large numbers. Second, to ensure the future use of the model, it could not require non-routine examinations that would incur additional expenses.

#### 2) Discussion resulting in discarding of variables

During additional rounds of discussion with the experienced clinicians (CW, DA), supplemented by several nurses and respiratory therapists (n = 8), the list of the remaining variables was re-evaluated.

#### 3) Panel discussion and adding of variables

Variables not currently on the list but considered by the expert panel (CW, DA) to be clinically important in decision-making were added. The roles of all the variables were further discussed and relationships among variables were established.

**Figure 1:**
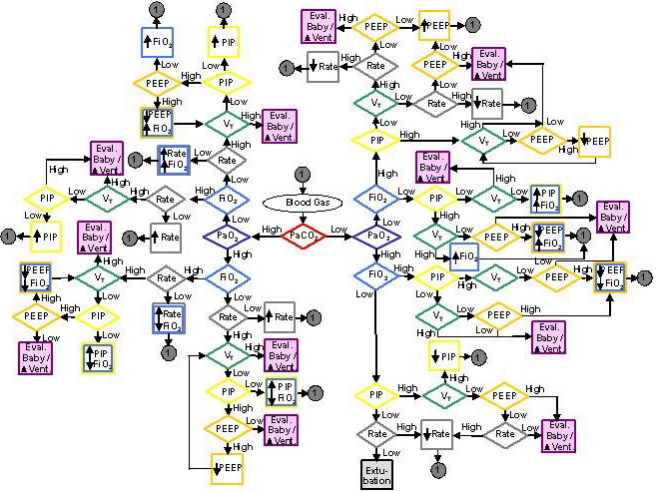
Example of an Algorithm for Ventilator Management (adapted from Carlo and Martin, 1986).

To visualize the relationships among some of the variables, we adapted a flow chart of the algorithm for ventilator management created by Carlo and Martin [17]. This flow-chart, shown in Figure 1, depicts the complexity of the relationships by illustrating potential actions (depicted as squares) that needed to be taken at a given blood gas analysis result (middle of graph). The goal of this chart is to reach the square labeled "extubate" in the middle bottom part that can be achieved through adjustments in the ventilator settings according to the results of the blood gas analysis. This graph is highly simplified, showing only the relationships between ventilator settings and blood gases. Furthermore, only binary (high versus low) situations for each decision node (diamonds) were considered. Inclusion of a third possibility, such as "adequate" would require a third dimension, and any inclusion of more than 3 possibilities would render it impossible to visualize the relationships.

#### 4) Bedside visits and adding of variables

In this step, two expert clinicians (CW, DA) were followed regularly during bedside visits in the NICU. The clinicians were encouraged to "think out loud" as suggested by Ericsson and Simon [18] while determining the status of a given infant and assessing the possibility for extubation. These authors stated that "an explanation of thoughts, ideas, or hypotheses or their motives is not simply a recoding of information already present in STM [short term memory], but requires linking this information to earlier thoughts and information attended to previously". Through this process, we were able to develop a chart listing the variables that were considered during the assessment of a given infant. Any variable that was mentioned during the bedside visit was included in the chart regardless of perceived subjective importance for extubation. All variables were grouped into one of nine categories: baseline assessments, demographics, vital signs, weight, ventilator settings, age, medication, "baby looks comfortable", and blood gases.

#### 5) Discussion about discarding of variables and inclusion of new variables

In further discussions with the clinicians (CW, DA), qualitative variables, such as "baby looks comfortable" or "work of breathing" were translated into new variables composed of quantitative parameters previously identified. Further, combinations of several parameters were used to create new variables such as "over ventilated" or "balanced pattern", which looks at a balanced pattern of ventilator settings. (For example, the infant may have weaned to room air (i.e. FiO_2 _= 21%) but is still requiring a high peak inspiratory pressure to maintain oxygenation.) After data collection, variables were discarded if their values were zero for at least 90% of the infants included in the study.

The two clinical experts (CW, DA) and two additional neonatologists (MS, JC) were provided with the complete list of variables and were asked to rate each variable on a scale from 0 (not important) to 10 (very important) for its relevance in their decision-making whether or not to extubate a given infant. The resulting ratings were used to determine the mean score for each variable and its relative importance compared to the other parameters.

In the final phase, we used the complete set of variables to develop an ANN and a multivariate logistic regression (MLR) model (see below) to determine the best performing model of each category. Extensive modeling was carried out to determine the best performing ANN with the optimal set of predictive variables. In parallel, multiple MLR models were developed to determine the optimal variable set for this method. The best performing models of the two methods were then compared using sensitivity analysis and ROC curves (for details see [11]).

To explore further the diverse approaches – the two computational models and clinical expertise – we compared the ranking of each variable, i.e. their relative importance in the full set across the three methods (ANN, MLR, CE) using Wilcoxon Signed Rank Test.

### Artificial neural network

ANNs are a machine-learning technique modeled after natural nervous systems. In accordance with the structure of the nervous system, the units in an ANN discriminant function called neurons or nodes are arranged in layers: the outer layers containing the input and output nodes and at least one inner layer of hidden nodes where the processing takes places. Nodes are fully interconnected between layers but, typically, not within them. All connections between nodes carry weights, thus increasing or decreasing the relative importance of certain nodes. The regression process of repeatedly adjusting these weights is designated as learning, which is stopped when the resulting error function is minimized, that is, the difference between the observed outcome and the calculated output [19]. Cross-validation, i.e. using subsamples of the data for training while retaining other subsamples for validation with unseen data, was used for early stopping of the ANN regression. Combined with bootstrapping, which resamples with replacement from the original sample of the number of hidden nodes, this method enables ANN predictors to identify signals from noisy and unbalanced sampled data [20,21]. Almeida provides a recent review of ANN for the analysis of complex biomedical data [22].

MLR, a well-established statistical modeling tool [23], uses sigmoidal functions for multiple parameters to discriminate among categories. ANN identification is analogous to two consecutive logistic regressions; therefore, the multi-layered ANN topology in this study uses sigmoid transfer functions. The fact that ANNs generalize MLR to accommodate non-linear dependencies makes the expectation of better performance of ANN over MLR mostly an issue of proper implementation. Nevertheless, the relative novelty of neural computing argues for a comparative study of ANN and MLR.

For the comparison of the ANN approach with MLR and clinical expertise, a series of 10 ANNs using 51 input variables was developed. Because random values are assigned to the weights of the nodes at the start of the training of a given ANN, the results for these 10 ANNs were expected to differ. For each of the 10 ANNs, we performed a sensitivity analysis that determines the influence of each input variable on the output of the ANN, the prediction score. Variables with high sensitivities are considered most important in the model for the prediction. The mean sensitivity for each variable across all 10 ANNs was calculated and ranked from largest to smallest.

### Multivariate logistic regression

This method models a dichotomous or ordinal outcome such as extubation success or failure as a function of a set of independent (predictor) variables. All 51 variables, developed as part of the initial list of potential variables, also were considered in the multivariate logistic regression analysis. In the development of the first MLR model (MLR model 1), the 51 variables were entered along with the first order interaction terms among all 51 variables and the second order interaction terms for variables known *a priori *(clinically) to interact. In a second model (MLR model 2), first order interaction terms of variables with correlations below an arbitrarily chosen cut-off point (ρ = |0.6|) were entered into the model along with the 51 variables and known clinical interactions. In a third model (MLR model 3), interaction terms of variables with correlations above |0.6|) were entered into the model along with the 51 variables and known clinical interactions (see additional file 1). These models were subjected to forward selection procedures. The resulting regression models were compared for their goodness-of-fit as determined by Akaike information criterion and Hosmer and Lemeshow goodness of fit test and then evaluated for their predictive capabilities by determining the receiver operating characteristic (ROC) curves and area under the curve (AUC) for the predictions using the validation data. The model with the largest AUC for the validation set was used for comparison with the ANN and clinical experts.

To determine the relative importance of the variables of the MLR model, we used the χ^2 ^values for both sets of variables: the variables included in and the variables excluded from the final model. The variable with the largest chi-square value (and the most significant p-value) received the highest score; the variable with the smallest chi-square value (and the least significant p-value) received the lowest score for relative importance.

To further compare the ANN and the MLR computational models to the clinical experts (CE), we used the sensitivity values for the individual variables returned by the ANN, the chi-square values for the MLR and the mean rank for the CE (CW, DA, MS, JC) as importance scores. Relative importance determined from these importance scores was used to compute Wilcoxon Signed Rank Test statistics for comparison of the three methods. P-values were Bonferroni-adjusted for multiple comparisons.

### Receiver operating characteristic curves

ROC curves examine the performance of a given computational model [25]. True-positive and false-positive predictions are compared against a range of decision thresholds. A true-positive prediction classifies an infant who remains extubated as an extubation success. A false-positive prediction classifies an infant who has failed extubation as having success. The probability of a true-positive result refers to the sensitivity of the prediction model. The probability of a false-positive result is designated as 1-specificity, where specificity is the proportion of times the model will classify an infant as failure when he has failed extubation. In a coordinate system, the ROC curve graphically shows the performance of the network with sensitivity representing the y-axis and 1-specificity on the x-axis (i.e. the true-positive rate is plotted versus the false-positive rate for the prediction model). The area under the curve (AUC) is equivalent to the probability that a successfully extubated infant will be considered to be more likely an extubation success than a failure, or an infant who failed extubation will be considered to be more likely an extubation failure than a success [26]. The results of the sensitivity analysis and the ROC curves for the ANN were compared to those obtained using logistic regression.

### Sensitivity analysis

This methodology assesses the effect of change in input variables on the output [22]. The sensitivity to a given input variable is quantified by the ratio between the change (1^st ^derivatives) in output value over the change in input value that causes it (equation 2) multiplied by 100.

### Novelty index

The training of ANN is implemented with cross-validation for early stopping to avoid over-fitting [22]. However, there are no guarantees that predictions for new data will be valid if those data are not within the original domain of values. The purpose of the novelty index is to quantify the degree of novelty of a new submission for ANN prediction. Predictions generated for submissions classified as "novel" should be considered cautiously or ignored altogether.

#### Rationale

The novelty measure developed below combines the Euclidean distance, ED, between a newly submitted set of parameters and the reference data set (equation 1) with the predictive sensitivity to the individual parameters (equation 2). By use of the weighted Euclidean distance (WED), the novel index obtained (equation 3) will account for the importance of a given parameter in the context of the values of the other parameters – e.g., an outlying value of a parameter to which the prediction has low sensitivity should count less than that of a parameter with high sensitivity.

#### Method development

The novelty of a new submission, y, could conceivably be obtained simply by comparing the new values with the reference values, x, (the ones used to train the ANN). A straightforward approach would be to measure Euclidean distance (equation 1) between the new submission, y, and the reference set, x, and compare them with the distances within the reference set.

ED(x,y)=∑i=1#parameters(yi−xi)2     (1)
 MathType@MTEF@5@5@+=feaafiart1ev1aaatCvAUfKttLearuWrP9MDH5MBPbIqV92AaeXatLxBI9gBaebbnrfifHhDYfgasaacH8akY=wiFfYdH8Gipec8Eeeu0xXdbba9frFj0=OqFfea0dXdd9vqai=hGuQ8kuc9pgc9s8qqaq=dirpe0xb9q8qiLsFr0=vr0=vr0dc8meaabaqaciaacaGaaeqabaqabeGadaaakeaacqWGfbqrcqWGebarcqGGOaakcqWG4baEcqGGSaalcqWG5bqEcqGGPaqkcqGH9aqpdaGcaaqaamaaqahabaWaaeWaaeaacqWG5bqEdaWgaaWcbaGaemyAaKgabeaakiabgkHiTiabdIha4naaBaaaleaacqWGPbqAaeqaaaGccaGLOaGaayzkaaWaaWbaaSqabeaacqaIYaGmaaaabaGaemyAaKMaeyypa0JaeGymaedabaGaei4iamIaemiCaaNaemyyaeMaemOCaiNaemyyaeMaemyBa0MaemyzauMaemiDaqNaemyzauMaemOCaiNaem4CamhaniabggHiLdaaleqaaOGaaCzcaiaaxMaadaqadaqaaiabigdaXaGaayjkaiaawMcaaaaa@5701@

However, this approach would not take into consideration that the difference in parameter values accounting for the distance corresponds to parameters with variable predictive sensitivity (equation 2).

SO←yi=dyidO.Oyi     (2)
 MathType@MTEF@5@5@+=feaafiart1ev1aaatCvAUfKttLearuWrP9MDH5MBPbIqV92AaeXatLxBI9gBaebbnrfifHhDYfgasaacH8akY=wiFfYdH8Gipec8Eeeu0xXdbba9frFj0=OqFfea0dXdd9vqai=hGuQ8kuc9pgc9s8qqaq=dirpe0xb9q8qiLsFr0=vr0=vr0dc8meaabaqaciaacaGaaeqabaqabeGadaaakeaacqWGtbWudaWgaaWcbaGaem4ta8KaeyiKHWQaemyEaK3aaSbaaWqaaiabdMgaPbqabaaaleqaaOGaeyypa0ZaaSaaaeaacqWGKbazcqWG5bqEdaWgaaWcbaGaemyAaKgabeaaaOqaaiabdsgaKjabd+eapbaacqGGUaGldaWcaaqaaiabd+eapbqaaiabdMha5naaBaaaleaacqWGPbqAaeqaaaaakiaaxMaacaWLjaWaaeWaaeaacqaIYaGmaiaawIcacaGLPaaaaaa@4500@

*O *value of output parameter, calculated as an ANN(y)

y_*i *_value of i^th ^input parameter

SO←yi
 MathType@MTEF@5@5@+=feaafiart1ev1aaatCvAUfKttLearuWrP9MDH5MBPbIqV92AaeXatLxBI9gBaebbnrfifHhDYfgasaacH8akY=wiFfYdH8Gipec8Eeeu0xXdbba9frFj0=OqFfea0dXdd9vqai=hGuQ8kuc9pgc9s8qqaq=dirpe0xb9q8qiLsFr0=vr0=vr0dc8meaabaqaciaacaGaaeqabaqabeGadaaakeaacqWGtbWudaWgaaWcbaGaem4ta8KaeyiKHWQaemyEaK3aaSbaaWqaaiabdMgaPbqabaaaleqaaaaa@3425@ sensitivity of predicting O from y_i_

Consequently, we have used a novelty index that compares distances weighted for the corresponding sensitivities (3):

WED(x,y)=∑i=1#parameters(SO←yi⋅yi−SO←xi⋅xi)2     (3)
 MathType@MTEF@5@5@+=feaafiart1ev1aaatCvAUfKttLearuWrP9MDH5MBPbIqV92AaeXatLxBI9gBaebbnrfifHhDYfgasaacH8akY=wiFfYdH8Gipec8Eeeu0xXdbba9frFj0=OqFfea0dXdd9vqai=hGuQ8kuc9pgc9s8qqaq=dirpe0xb9q8qiLsFr0=vr0=vr0dc8meaabaqaciaacaGaaeqabaqabeGadaaakeaacqWGxbWvcqWGfbqrcqWGebarcqGGOaakcqWG4baEcqGGSaalcqWG5bqEcqGGPaqkcqGH9aqpdaGcaaqaamaaqahabaWaaeWaaeaacqWGtbWudaWgaaWcbaGaem4ta8KaeyiKHWQaemyEaK3aaSbaaWqaaiabdMgaPbqabaaaleqaaOGaeyyXICTaemyEaK3aaSbaaSqaaiabdMgaPbqabaGccqGHsislcqWGtbWudaWgaaWcbaGaem4ta8KaeyiKHWQaemiEaG3aaSbaaWqaaiabdMgaPbqabaaaleqaaOGaeyyXICTaemiEaG3aaSbaaSqaaiabdMgaPbqabaaakiaawIcacaGLPaaadaahaaWcbeqaaiabikdaYaaaaeaacqWGPbqAcqGH9aqpcqaIXaqmaeaacqGGJaWicqWGWbaCcqWGHbqycqWGYbGCcqWGHbqycqWGTbqBcqWGLbqzcqWG0baDcqWGLbqzcqWGYbGCcqWGZbWCa0GaeyyeIuoaaSqabaGccaWLjaGaaCzcamaabmaabaGaeG4mamdacaGLOaGaayzkaaaaaa@6BD4@

The comparison between WED's and the most similar reference entry (min(WED(x,y))) is performed to determine the novelty index (NI):

NI=min⁡(WED(x,y))median(min⁡(WED(x,x)))     (4)
 MathType@MTEF@5@5@+=feaafiart1ev1aaatCvAUfKttLearuWrP9MDH5MBPbIqV92AaeXatLxBI9gBaebbnrfifHhDYfgasaacH8akY=wiFfYdH8Gipec8Eeeu0xXdbba9frFj0=OqFfea0dXdd9vqai=hGuQ8kuc9pgc9s8qqaq=dirpe0xb9q8qiLsFr0=vr0=vr0dc8meaabaqaciaacaGaaeqabaqabeGadaaakeaacqWGobGtcqWGjbqscqGH9aqpdaWcaaqaaiGbc2gaTjabcMgaPjabc6gaUjabcIcaOiabdEfaxjabdweafjabdseaejabcIcaOiabdIha4jabcYcaSiabdMha5jabcMcaPiabcMcaPaqaaiabd2gaTjabdwgaLjabdsgaKjabdMgaPjabdggaHjabd6gaUjabcIcaOiGbc2gaTjabcMgaPjabc6gaUjabcIcaOiabdEfaxjabdweafjabdseaejabcIcaOiabdIha4jabcYcaSiabdIha4jabcMcaPiabcMcaPiabcMcaPaaacaWLjaGaaCzcamaabmaabaGaeGinaqdacaGLOaGaayzkaaaaaa@5AF1@

*x *is the reference dataset. Distances between x and x will result in the cross-tabulation of distances

*y *is the vector of new values. Distance between x and y is a vector with as many elements as rows in the reference dataset.

min(*WED*) is the closest reference input

The novelty index ranges from 0 to infinity; values markedly above one indicate the degree of novelty of the new set of input parameters.

### Web-interface

**Figure 2:**
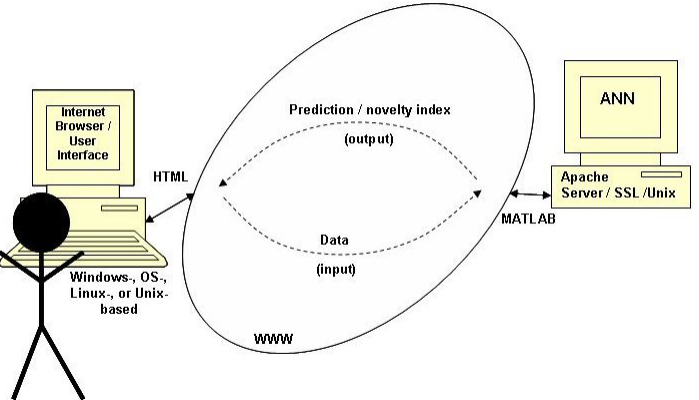
**Schematic representation of the prediction tool**. Data entered by the user are delivered through the Internet to the ANN housed on an Apache server. The ANN that was programmed in MATLAB calculates the prediction, which is again returned through the Internet to the user and displayed in the Internet browser.

Using the above results, a decision-support tool was developed consisting of two components: the web-based user interface and the mathematical model used to determine the prediction and reliability measures. Application deployment is entirely on the server side, housed on a dual-processor computer, configured as a webserver, at the Medical University of South Carolina (Figure 2). The interface is generated as Hypertext Markup Language (HTML), which is OS-independent and can be accessed through any web browser. Data entered through the interface are submitted to the ANN (see below).

The ANN was implemented in the MATLAB scientific computing programming environment (27); secure access is configured through an Apache Linux server running Secure Socket Layer (SSL) with 128-bit encryption. The ANN processes the new input provided from the user to calculate the predicted outcome for a given infant. This calculation is based on data used for the development, training and validation of the ANN. Simultaneously, the input data are compared to the training data to determine how similar or different the new data are and the novelty index is computed (see below). Furthermore, a sensitivity analysis is carried out to determine the importance of each input variable for the output. The prediction score and the novelty index are delivered back to the HTML script and displayed in the user interface along with the ROC curve and a table of sensitivity/specificity pairs (see below) generated after training and validation of the ANN. The development and training of the ANN model were presented in detail in a previous report by the authors [11].

### Study population

Data collection was based on an Institutional Review Board-approved retrospective chart review of medical records of infants born between January 1999 and October 2002. These data have been published previously in a detailed report describing the development and comparison of the ANN, MLR, and CE models [11]. To ensure a satisfactory sample size, we included very low birth weight (VLBW) infants (1,000–1,500 g) and extremely low birth weight infants who weighed between 900–1,000 g. Because birth weight may vary greatly for infants at a comparable gestational age, selecting a sample based on gestational age could result in a wide spread of birth weights. Conversely, infants with birth weights less than 900 g are extremely immature and, therefore, clinically distinct from infants with larger weights [28]. Similarly, infants with birth weights larger than 1,500 g are generally less premature, and therefore, less likely to develop RDS. Thus, to be included in the study, infants had to have a birth weight between 900 and 1,500 g and a diagnosis of RDS. The infants were intubated and managed on mechanical ventilation within 6 hours after birth. Data were collected from time of intubation to the first attempt of extubation. An extubation attempt was decided by the medical team under the supervision of a board-certified attending neonatologist. Table 1 gives an overview of the parameters that needed to be in place prior to an extubation attempt. Those infants who had an unintentional extubation, that is became extubated without a medical decision for extubation, were followed if they remained extubated beyond the immediate extubation. Infants who had evidence of respiratory failure after extubation despite nasal continuous positive air pressure (CPAP) within 48 hours after extubation were reintubated and considered an extubation failure. Because it is well known that infants who are extubated to nasal CPAP are less likely to have respiratory deterioration, it is the usual practice of this NICU to extubate to CPAP, although this was not a rigidly defined practice during the study period. Reintubation was based on clinical criteria set forth by the clinical team that included increased work of breathing and respiratory failure based on rising pCO_2 _and pH below 7.25 and repeated apneic and bradycardic events unresponsive to methylxanthine therapy or nasal CPAP.

**Table 1:** Guidelines for an Extubation Trial

Infant	with spontaneous respiratory effort above set ventilator rate
	without evidence of respiratory failure (such as intercostals retractions, poor chest wall movement) and respiratory rate <80 breaths/minute
	with apneic or bradycardic events that decreased in severity and frequency following initiation of methylxanthine therapy
Ventilator rate	≤20 breaths/minute
FiO_2 _requirement	<40%
Blood gas parameters in the normal range	for arterial blood gas determinations: pH ≥ 7.25 pCO_2 _<60–64
	for capillary blood gas determinations: pH ≥7.2 pCO_2 _<70
Saturation (SaO_2_)	consistently >90%

## Results

A total of 183 infants who fulfilled the eligibility criteria were randomly assigned to two independent data sets, one set using ~70% of the total sample for development and training of the neural network (n = 130) and the second set using ~30% of the total sample for testing (validation) purposes (n = 53).

Twenty-seven infants were excluded from the study for the following reasons: pulmonary hypertension (n = 1); time between delivery and intubation >6 hours (n = 12); extubation from ventilators other than assist-control or synchronized intermittent mandatory ventilation (SIMV) (n = 5); life-support withdrawn without any previous attempt to extubate (n = 4); or no respiratory data retrievable from medical records (n = 6).

**Figure 3:**
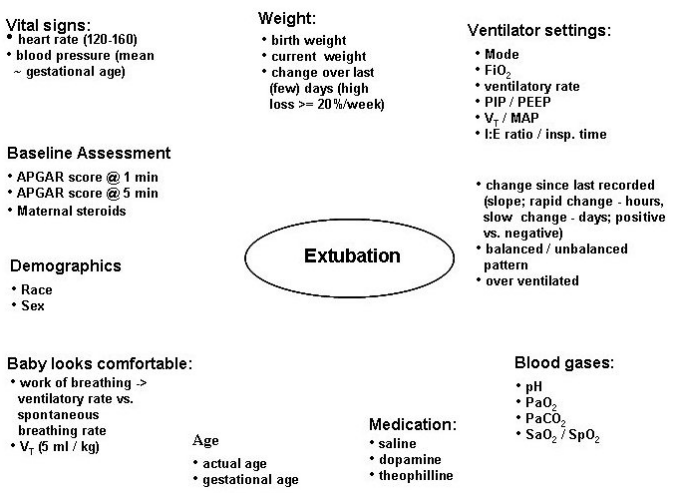
Variables Relevant for Outcome Prediction.

A total of 53 variables in nine categories were identified to play some role in the decision whether or not to extubate a premature infant who is mechanically ventilated (Figure 3). These included demographic variables (such as gestational age, gender, race, and birth weight), APGAR scores, ventilator settings, blood gas analysis results, and vital signs (for complete list of variables, see appendix, Tables 2 and 3). Several indices assessing different physiological functions that had been examined in previous studies were considered for their potential to predict extubation outcome [2,6,29,30]. However, because the data were collected retrospectively, variables were required to be part of routine evaluation of the infant to be included. Consequently, only variables easily available at the bedside were considered. For variables recorded at routine evaluations, such as ventilator settings, blood gas analysis results, and vital signs, data from the two time points immediately prior to extubation were included. Two variables (dopamine use, >20% weight change) were discarded after data collection because more than 90% of all values for the two variables were zero. Almost half (45%) of the infants were extubated within 24 hours of their birth, and therefore, their current weight equaled their birth weight. Approximately 19% of the infants were extubated after 5 days of life and had current weights markedly different from their birth weights. Therefore, birth weight was dropped and only current weight was used further. For modeling purposes, the variable race was coded as two dummy variables: Caucasian 0/1 and African-American 0/1. The remaining 51 variables were all considered potentially predictive and included in further analyses using ANN and MLR models.

**Table 2:** Range for total sample and explanations of variables used

Variable	Minimum	Maximum	Explanation
Age_D	1	31	Age in days (~ = time on ventilator)
APGAR_1	0	9	APGAR Score at 1 minute
APGAR5_1	-2	7	Difference between APGAR at 1 and 5 minutes
BE	-7.5	6	Base excess content in blood
BP	28.5	67	Blood pressure
CurrWeight	808	1600	Current weight
dBE	-4.5	1.7	Change in base excess since last measurement
dBP	-13.7	27.0	Change in blood pressure since last measurement
dFIO_2_	-30.0	21.8	Change in FiO_2 _since last measurement
dHCO_3_	-3.0	4.0	Change in HCO_3 _since last measurement
dIErat	-3.5	2.8	Change in I:E ratio since last measurement
dINSP	0.0	1.0	Change in INSP since last measurement
dMAP	-2.4	1.1	Change in MAP since last measurement
dPaCO_2_	-6.4	22.0	Change in PaCO_2 _since last measurement
dPaO_2_	-63.3	50.0	Change in PaO_2 _since last measurement
dPEEP	-3.0	1.3	Change in PEEP since last measurement
dPH	-0.2	0.1	Change in pH since last measurement
dPIP	-3.0	2.4	Change in PIP since last measurement
dPulse	-27.0	42.0	Change in heart rate since last measurement
dRATE	-15.0	10.6	Change in ventilatory breathing rate since last m.
dRRatio	-3.3	3.4	Change in rate ratio (spont. vs. vent.) since last m.
dSaO_2_	-4.0	15.0	Change in SaO_2 _since last measurement
dTIME	0.3	51.6	Time between last two blood gases analyses
dV_*T*_	-1.8	2.5	Change in V_*T*_
FiO_2_	20	60	Oxygen content in air delivered by ventilator
Gst_age	25	35	Gestational age
HCO_3_	16	35	Bicarbonate content in blood
IEratio	1	17.2	I : E ratio (inspiratory to expiratory time)
INSP	0.23	0.5	Inspiratory time
Lag	0.25	17.5	Lag time between last blood gas result and extubation
MAP	4.2	10.9	Mean airway pressure
PaCO_2_	23	65	Partial pressure of carbon dioxide in blood
PaO_2_	23	228	Partial pressure of oxygen in blood
PEEP	3	5	Positive end-expiratory pressure
pH	7.24	7.58	Acidity or alkalinity of blood
PIP	10	21	Peak inspiratory pressure
Pulse	112	187	Heart rate
R_ratio	0.4	6.0	Ratio: spontaneous breathing rate/ventilatory rate
Rate	10	60	Ventilatory breathing rate
Saline	0	26	Saline bolus within 24 hours prior to extubation
SaO_2_	79	100	Oxygen saturation in blood
Theoph	0	8.3	Theophilline bolus within 24 hours prior to extubation
V_*T*_	3	15.7	Tidal volume
TXBETAME	0	1	Maternal betamethasone

**Table 3:** Frequency for total sample and explanation of variables used

Variable	Frequency (n = 183)	Proportion
AB (Arterial blood gas)	125	68.3%
AB (Cap. blood gas)	58	31.7%
Balanced Pattern (no)	115	62.8%
Balanced Pattern (yes)	68	37.2%
Extubation Failure	35	19.1%
Extubation Success	148	80.9%
Mode (AC)	38	20.8%
Mode (SIMV)	145	79.2%
Overventilated (no)	88	48.1%
Overventilated (yes)	95	51.9%
Race (Black)	67	36.6%
Race (Other)	6	3.3%
Race (White)	110	60.1%
Sex (Female)	87	47.5%
Sex (Male)	96	52.5%

Descriptive characteristics were similar in the two data sets (Table 4). Mean (± standard deviation) gestational age for the training set was 29.3 weeks (± 2.0) compared to 29.1 weeks (± 2.0) for the validation set. The mean birth weight was 1,170 grams (± 168) for the training set and 1,145 grams (± 182) for the validation set. Median time on ventilator was 2 days for both the training and the validation sets.

**Table 4:** Demographic characteristics of subject population (mean ± SD)

	Total set (n = 183)	Training set (n = 130)	Validation set (n = 53)	p-value
Gestational age (weeks)	29.3 ± 2.0	29.3 ± 2.0	29.1 ± 2.0	0.471
Birth weight (grams)	1164 ± 172	1170 ± 168	1145 ± 182	0.391
Time on ventilator (~Age in days) (median)	2	2	2	--
Sex				

Male	52.5% (96/183)	52.3% (68/130)	52.8% (28/53)	0.953
Race				

Caucasian	60.1% (110/183)	62.3% (81/130)	54.7% (29/531)	0.233
African-American	36.6% (67/183)	33.1% (43/130)	45.3% (24/53)	0
Hispanic	2.7% (5/183)	3.8% (5/130)	0	--
Asian	0.5% (1/183)	0.8% (1/130)	0	--
Outcome				

Extubation Failure	19.1% (35/183	16.9% (22/130)	24.5% (13/53)	0.243

^1^Pooled t-test; ^2^t-test for unequal variances; ^3^Chi-square test

Missing data were found only in the ventilator setting recordings. Deleting all cases with missing data would not only significantly reduce the sample size but also lead to a potentially biased data set and result in the loss of potentially valuable information [31]. A total of 74 infants had missing values for at least one of the following variables: mean airway pressure (MAP), inspiratory time (INSP), ratio of inspiratory to expiratory time (I:E ratio), and tidal volume (V_T_). For those infants (n = 72) for whom the MAP value closest to extubation was missing, we estimated the MAP from previously measured values using the equation described by Carlo and Martin [17]. In 52 cases for which INSP, I:E ratio and V_T _values were missing closest to extubation, last observation carried forward (LOCF) was used to impute the missing values. For two infants no previous values were available for any of the 4 variables; therefore, overall means were used to impute missing values. Potential bias resulting from imputation was considered less severe than the loss of data due to the exclusion of infants with missing data.

The two data sets (training and validation) containing the 51 variables were used to develop and compare the mathematical models (described in detail in [11]). After extensive modeling for the ANN as well as the MLR methods, the best performing median ANN model, corresponding to the topology with the best median ANN consisted of 3 layers with 13 input nodes in the first layer, 7 hidden nodes in the second layer, and a single output node, corresponding to the prediction of extubation outcome. The final ANN included 13 variables in 4 categories: pH, SaO_2_, PaO_2_, peak inspiratory pressure (PIP), positive end-expiratory pressure (PEEP), MAP, V_T_, INSP, I:E ratio, ventilator mode (mode), pulse, blood pressure (BP), and gestational age. In contrast, the best MLR model contained 4 variables in 3 categories, 3 of the 13 variables also were used by the ANN model (V_T_, PaCO_2_, ventilator mode) and one variable was new (race: 1 = African-American). The equation for the best MLR model can be written as follows: logit(θ) = 3.38 + 0.364X_1 _-0.114X_2 _+ 0.542X_3 _- 0.659X_4 _with X_1 _= tidal volume (V_T_), X2 = PaCO_2_, X_3 _= (1 if African-American; 0 otherwise), X4 = ventilator mode. The area under the ROC curve for the ANN was 0.87 and 0.81 for validation and training sets respectively, compared to 0.75 (validation) and 0.81 (training) of the MLR model. For the four clinical experts (CE) sensitivity/1-specificity pairs were obtained (corresponding to the validation of ANN/MLR), ranging from 0.68/0.29 to 0.98/0.83. No single parameter was as predictive of outcome as the combined 13 variables in the final ANN.

Comparison of the ranking of all 51 variables for the 3 models using the Wilcoxon Signed Rank Test showed that whereas the relative importance of the 51 variables in the MLR was not significantly different from that of either the ANN (p = 0.726) or the CE (p = 0.306), the ranking of the variables in the ANN was found to be significantly different (p = 0.0018) from the ranking of the variables performed by the CE. While pH was considered the single most important variable in the ANN model and among several variables with higher scores for importance by the clinicians, it was found among the least important variables of the MLR. PaCO_2 _was considered the most important variable in the MLR; in the ANN and the CE model it was ranked among the more important variables. Tidal volume and ventilation mode, also considered very important for the MLR, were considered of similar importance by the CE and ANN models, but much lower than the MLR.

**Figure 4:**
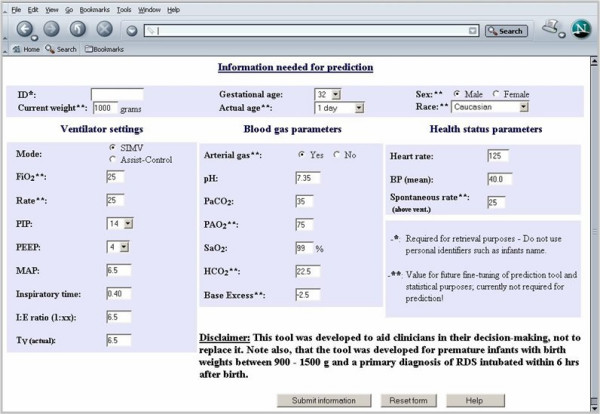
**Data Entry Page for Decision-Support Tool**. Thirteen variables are required for calculation of the prediction. Additional variables are requested for statistical purposes and future fine-tuning of the prediction tool. Once all required fields are completed, the information is submitted to the ANN by clicking the appropriate button.

The ANN, which was considered the most predictive model, was further used in the implementation of a decision-support tool on the Internet. Upon accessing the decision-support tool called "The Premies Project" [32] and following the link for the prediction, the web-page for data entry is displayed (Figure 4).

The ANN currently employed in the prediction system uses 13 input variables identified as most predictive to determine the prediction score, including gestational age, several ventilator parameters, blood gas analysis results, and vital signs. Several additional reference variables that are not used to compute the predictions are requested from the clinician: 1) the ID variable is required for future storage and retrieval purposes and ensures that the actual extubation outcome reported by the clinician can be matched to the appropriate input variables; and 2) the demographic variables (actual age of the infant, sex, race, and current weight) as well as two additional ventilator variables (FiO_2 _and breathing rate), two blood gas variables (bicarbonate and base excess), and spontaneous breathing rate are not required for the prediction. These variables are needed solely for statistical purposes, such as the comparison of the infant population used for the development of the ANN with the population for which predictions were calculated. However, the additional variables may be needed for future improvement of the prediction tool, namely as it applies to extending the domain of ANN training.

**Figure 5:**
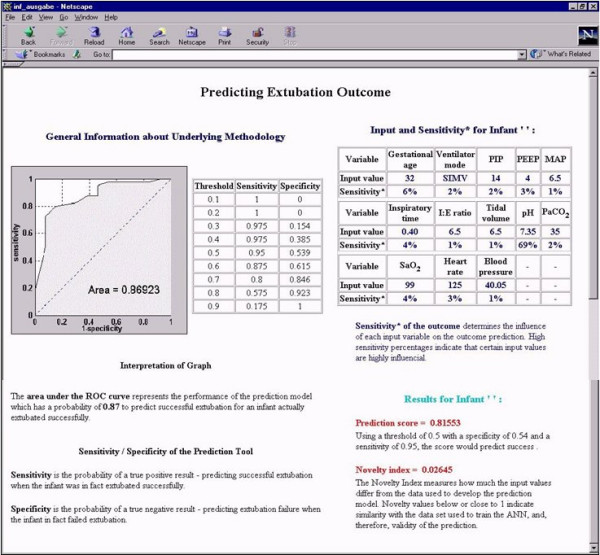
**Results page of decision-support tool**. The ANN returns the prediction score along with the novelty index. For categorization of the prediction into success or failure, a table with threshold values and the appropriate sensitivity/specificity pairs is provided. The novelty index indicates the level of confidence in the prediction.

Once the information for a given infant has been entered into the website by e.g. the clinicians, nurse, or respiratory therapist, it can be submitted by clicking on the button in the bottom part of the screen. The data are sent to the ANN, which calculates the prediction along with the sensitivities and the novelty index. The results are returned to the user and displayed using the HTML interface (Figure 5).

To enable the clinician to make an informed decision, he/she is provided with several pieces of information simultaneously. In the left part of the screen, information about the performance of the ANN can be found in the form of a ROC curve and a table of sensitivity and specificity for a range of thresholds used to create the ROC curve. Explanations for both are provided onscreen along with the graph and table.

In the right part of the screen, the clinician finds the input variables and values used to determine the prediction. Underneath, the prediction score is shown along with an explanation of how to interpret the score to determine whether the score predicts extubation success or failure. Since different clinicians may have different preferences with respect to sensitivity and specificity, we provided the clinician with the possibility to choose the most appropriate threshold by providing the prediction score instead of a dichotomous result (success/failure) using a pre-specified fixed threshold. Furthermore, the clinician is provided with the results of the sensitivity analysis, which measures the importance of each input in the calculation of the prediction. High sensitivities indicate variables of high importance. Additionally, the novelty index is provided to inform the clinician about the reliability of the prediction. The closer the new input data provided to the ANN are to the data that were used for development of the ANN, the higher the confidence in the prediction. Values between 0 and 1 indicate high similarity between the new and the reference data, while values markedly above one indicate low similarity.

## Discussion

When Wulff [33] describes how to make the best decision in clinical decision theory, he states that clinicians base their decisions on recorded observations and acquired knowledge as well as evaluations of the consequences of their previous actions. However, clinicians are rarely able to explain the "algorithm" for their decisions, i.e. which variables were considered and how the variables were weighted in comparison with others, because large parts of the decision-making are subconscious processes. With the use of an expert panel and the 5-step procedure described in this report, a rather complex and time-consuming approach was used to distill 51 variables that incorporated all available knowledge about how the decision is made based on sound scientific reasoning.

These 51 potential predictive variables were then used to develop computational models for the prediction of extubation outcome using MLR and ANN modeling techniques. ANNs are advantageous because they can detect and model correlations among variables inherent in data. For possible inclusion of interactions between variables in MLR models, specific interaction variables have to be created, artificially increasing the number of variables and thus increasing the required sample size, and therefore, limiting the performance of a model developed using a smaller sample. Restricting interaction terms in the MLR to those with moderate to high multicollinearity could have put the MLR model at a disadvantage compared to the ANN due to a decrease in correct selections caused by an overlap of variance that the variables and their interactions account for in the model [34]. However, the model using interaction terms for highly correlated variables was found to be the best performing predictive MLR model. The relative advantage of ANNs is being less constrained by the shape of the interaction. The issue of parameterization in the ANN was addressed by use of variable selection to limit the number of interactions considered. The best performing ANN had the largest AUC (0.87) of all models developed. In comparison, the best MLR model achieved an AUC of 0.75. By use of sensitivity analysis, the discriminating dependency of ANNs can be explained and validated against the expertise of clinicians and traditional statistical methods, such as MLR. Sensitivity analysis assesses the effect of change in input variables on the output, and therefore, allows quantification of variables used to develop ANN models.

In the comparison of the full set of 51 variables among the three models, the ANN and the CE model seemed to agree more often than the MLR and the CE. For 53% of all variables considered, the ratings of percent importance in the models were closer for the ANN and CE model than for MLR and CE. Looking at the models separately, the CE considered approximately two-thirds (67%) of the variables as important for their decision, while the best ANN included 13 (25%) variables and the MLR used only 4 (8%). These results suggest that the ANN methodology was able to capture underlying thought processes of the clinicians from the data used for its development and training. However, the differences in ranking of some variables that were found to be significant may account for the better predictive performance of the ANN compared to the clinicians and suggest the need for further study.

The best performing ANN was further used for implementation of the decision-support tool. Our web-based interface in connection with the ANN prediction model can provide decision-support to clinicians in any NICU with the necessary hardware and Internet access. Internet-based prediction tools are sound investments because of the present ubiquity of computers with Internet connections in hospitals throughout the world and the inherent platform-independence afforded by Internet-based applications: no additional software is needed. Finally, the mathematical tool requires no special or additional skills other than handling the mouse and keyboard. Pull-down menus were used when possible to minimize typing effort as well as to minimize typing errors. Default values are displayed to clarify the format expected for data entry. Additionally, the interface was designed to be intuitive and easy to navigate. The interface is consistent with standard graphical user interfaces used, for example, in Windows- and Mac-based systems.

The decision-support tool was designed to grant a high degree of reliability by providing a novelty index and a sensitivity analysis along with the prediction score. The novelty index measures how much the data provided for the prediction differ from the data used for the development of the neural network. The sensitivity analysis determines the effect of the individual input variables on the output. These measures inform the clinician which variable had the largest impact on the prediction of extubation outcome for a given infant. Additionally, the plot of the ROC curve is displayed along with pairs of sensitivity and specificity for different thresholds. This added information allows the clinician to categorize the prediction score as success or failure according to the pair of sensitivity/specificity deemed most appropriate by the clinician. If a clinician prefers to decrease the number of false-positive decisions, sensitivity needs to be high, and a threshold of 0.5 or higher may be considered resulting in a sensitivity of at least 0.95. However, if both sensitivity and specificity are considered equally important and both numbers of false-positive and false-negative decisions should be limited at the same time, a threshold of 0.7 would be preferable with sensitivity of 0.8 and specificity of 0.85. While the dataset used for the development of this model is limited to infants with a birth weight of 900–1,500 g, we are currently investigating the model in an ongoing study of infants <900 g.

The ability of ANN to successfully predict the optimal extubation time for an infant, in this case, with RDS becomes a powerful tool for the clinician, particularly during high patient care census days and in teaching hospitals. The identification of key variables that are associated with successful extubation gives feedback to the clinician, with reinforcement of skills that recognize the patterns to predict successful extubation.

The clinical utility of such a process extends beyond the theoretical. The difficulty identifying criteria, which predict successful extubation in VLBW infants, may further be defined utilizing this model. That is, this model may form the basis for further research regarding successful extubation, perhaps extending the model's success rate. By identifying criteria which do not significantly change the likelihood of success, analysis of the decision-making process becomes easier. Additionally, education of clinicians also would benefit. Furthermore, the integration of blood gas and ventilatory data into an infant's electronic record creates a tool that can be applied in any NICU with Internet access.

## Conclusion

In summary, we were able to identify numerous variables considered relevant for the decision whether or not to extubate a mechanically ventilated premature infant with respiratory distress syndrome. We further used these variables to develop a web-based decision-support tool to aid clinicians in their decision. Through the use of the Internet, this prediction tool is easy to use, platform-independent, and easily accessible to clinicians throughout the world, and thus, adaptable to individual care centers and their specific patients.

## Competing interests

The author(s) declare that they have no competing interest.

## Authors' contributions

MM conceived of the study and its design, coordinated the study, carried out data collection, carried out model building and performed statistical analysis, and drafter the manuscript. CW conceived of the study, participated in its design and coordination, and helped draft the manuscript. DA participated in the study design and coordination, and helped draft the manuscript. TH participated in the study design and coordination, and helped draft the manuscript. RK participated in the study design and coordination, assisted in statistical analysis, and helped draft the manuscript. JA participated in the study design and coordination, assisted in model building, and helped draft the manuscript.

All authors read and approved the final manuscript.

## Pre-publication history

The pre-publication history for this paper can be accessed here:



## Supplementary Material

Additional File 1 **Interaction terms for MLR model – forward selection**. List of interactions used in the development of the final MLR model. Click here for file
