# Supplementary material for: Parameter selection for and implementation of a web-based decision-support tool to predict extubation outcome in premature infants
Source: BMC Med Inform Decis Mak. 2006 Mar 1;6:11. doi: 10.1186/1472-6947-6-11 (PMC1413521; doi:10.1186/1472-6947-6-11)
Supplement: Additional File 1 — Interaction terms for MLR model – forward selection. List of interactions used in the development of the final MLR model. [file 1472-6947-6-11-S1.DOC]

**Table 5. Interaction terms for MLR model – forward selection.**

| sex*white | Gender and race (1=Caucasian; 0 otherwise) |
| --- | --- |
| sex*black | Gender and race (1=African-American; 0 otherwise) |
| sex*white*black | Gender and race |
| gst_age*weight | Gestational age and current weight |
| PIP*PaCO2 | Peek inspiratory pressure and PaCO_2_ |
| rate*PaCO2 | Breathing rate and PaCO_2_ |
| PIP*MAP | Peek inspiratory pressure and mean airway pressure |
| FiO2*PaO2 | FiO_2_ and PaCO_2_ |
| PEEP *MAP | Positive end expiratory pressure and mean airway pressure |
| PaCO2*PEEP | PaCO_2_ and positive end expiratory pressure |
| PaO2*MAP | PaCO_2_ and mean airway pressure |
| PaCO2*TV | PaCO_2_ and tidal volume |
| TV*PIP | Tidal volume and peek inspiratory pressure |
| TV*PEEP | Tidal volume and positive end expiratory pressure |
| AB*PaCO2 | Blood gas (1=arterial, 0=otherwise) and PaCO_2_ |
| AB*PaO2 | Blood gas (1=arterial, 0=otherwise) and PaO_2_ |
| pH*HCO3 | pH and HCO_3_ |
| pH*BE | pH and base excess |
| pH*HCO3*BE | pH and base excess and HCO_3_ |
| ov*rate | Overventilated (1=yes, 0=no) and breathing rate |
| ov*rate*PaCO2 | Overventilated and breathing rate and PaCO_2_ |
| white*black | Caucasian and African-American |
| rate*mode | Breathing rate and ventilation mode |
| r_ratio*mode | Breathing rate ratio and ventilation mode |
| rate*r_ratio*mode | Breathing rate and breathing rate ratio and ventilation mode |
| ieratio*map | Inspiratory/expiratory time ratio and mean airway pressure |
| paco2*ph | PaCO_2_ and pH |
| ov*paco2 | Overventilated and PaCO_2_ |
| be*hco3 | Base excess and HCO_3_ |
| bal_pat*ov | Balanced pattern and overventilated |
| dpaco2*dph | Change in PaCO_2_ and change in pH |
| dhco3*dpaco2 | Change in HCO_3_ and change in PaCO_2_ |
